# Highly efficient narrowband terahertz pulse generated from a radial plasma oscillator via mid-infrared lasers

**DOI:** 10.1038/s41598-026-60292-5

**Published:** 2026-08-19

**Authors:** Jaeho Lee, Manoj Kumar, Dohyun Park, Bernhard Ersfeld, Dino A. Jaroszynski, Inhyuk Nam, Min Sup Hur

**Affiliations:** 1 Department of Physics, Ulsan National Institute of Science and Technology, Ulsan, 44919 Republic of Korea; 2 Department of Physics, Scottish Universities Physics Alliance and University of Strathclyde, Glasgow, G4 0NG UK

**Keywords:** Optics and photonics, Physics

## Abstract

Plasma-based terahertz (THz) sources are currently limited to low laser-to-THz conversion efficiencies. Generating narrowband THz radiation remains particularly challenging for high-power THz applications. Here we demonstrate an unprecedented 0.88% laser-to-THz energy conversion efficiency from the recently proposed radial plasma oscillator (RPO) scheme [M. Kumar et al., Phys. Rev. Lett. 134, 015001 (2025)] driven by mid-infrared laser pulses at $\:{\lambda\:}_{1}=4.0\:\,\upmu\:\mathrm{m}$ and $\:{\lambda\:}_{2}=5.416\:\,\upmu\:\mathrm{m}$. Theoretical modelling and quasi-3D particle-in-cell simulations have been used to achieve THz pulse energies up to 0.341 mJ, with a peak field strength of 6.94 GV/m and a narrow spectral width ~ 15.5%. It is also shown that the high efficiency is well-preserved under field-ionized and collisional plasma conditions, making it a practical pathway toward compact narrowband high-power THz sources.

## Introduction

Recent advances have highlighted the usefulness of intense THz waves for biomedical applications, THz-driven particle accelerators, and ultrafast spectroscopy of large molecules (nucleic acids, proteins, etc.)^1–9^. Frequency-tuneable, narrow bandwidth THz sources producing GV/m-level fields will enable nonlinear material studies^10–12^ and act as drivers of GeV-scale compact accelerators^13–15^. For a THz source to be a competitive option as a driver for these applications, a high efficiency in laser-to-THz conversion is a key requirement, as it determines whether the high-field-strength THz radiation can be realized from a compact system. Conventional solid-state THz generation methods, such as optical rectification in nonlinear crystals^16,17^, offer efficiencies of a few percent level, but their maximum output powers of the THz emission are limited by the material damage threshold. Unlike the crystals, plasma can be a promising alternative for a radiating medium^18–26^, because the plasma is inherently resistant to extreme driving laser fields (~ TV/m). However, the plasma-based THz sources are often constrained by a low energy conversion efficiency. In particular, the efficiencies of plasma-based, *narrowband* THz sources are typically $\:{10}^{-3}-{10}^{-2}\:\%$^27–37^. This limitation originates from the inefficient coupling of *travelling* plasma wave and electromagnetic emission from that.

**Fig. 1:**
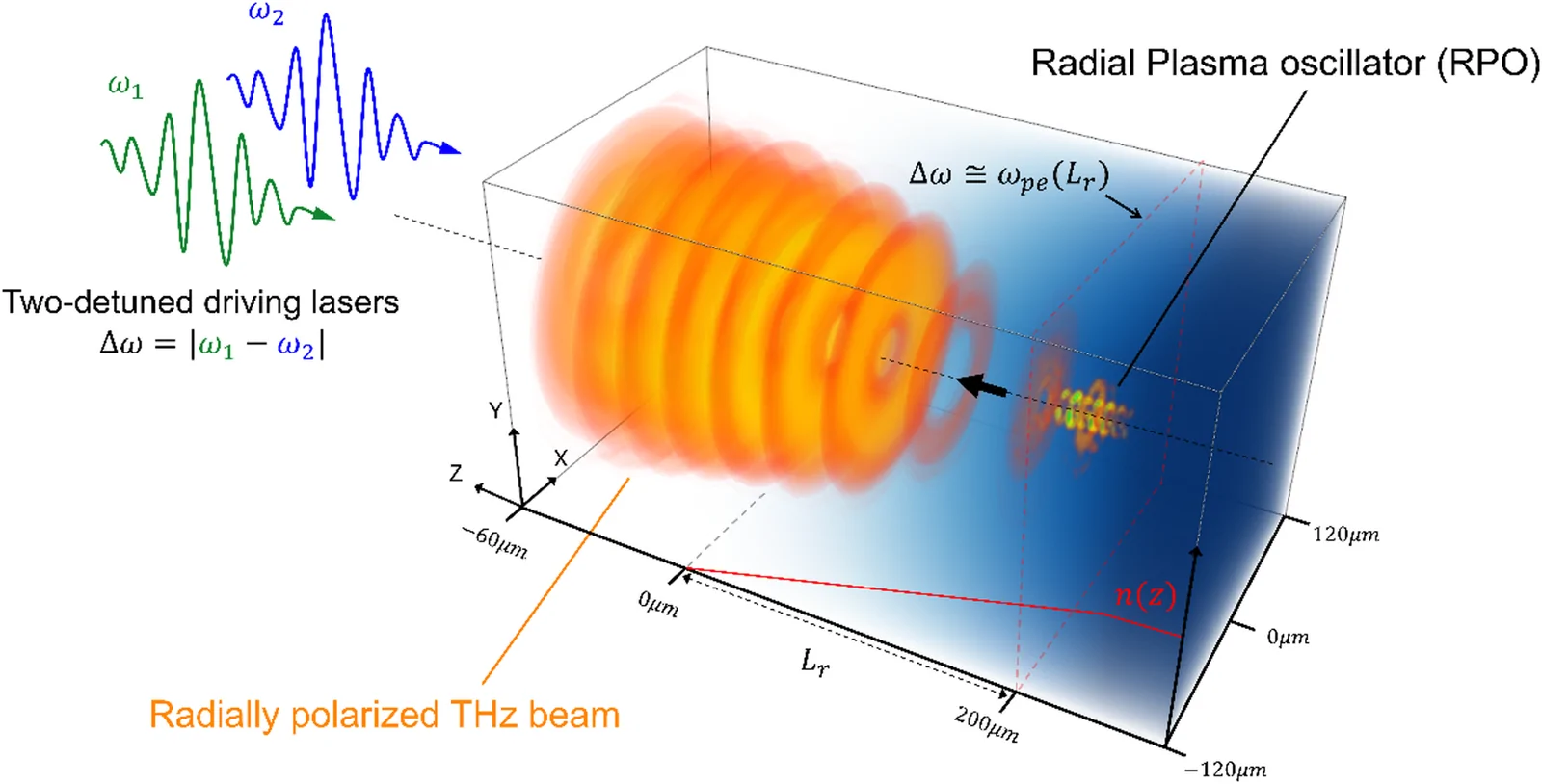
Schematic of a radial plasma oscillator (RPO) embedded in a density gradient plasma. The RPO is generated at the resonance position, where the beat frequency of driving laser pulses matches with local plasma frequency, $\:{\Delta\:}\omega\:\cong\:{\omega\:}_{pe}\left({L}_{r}\right)$. The RPO acts as a planar antenna, radiating a highly directional, radially polarized THz beam propagating backward along the density gradient. The blue region represents the density gradient plasma with its longitudinal profile displayed on the front wall by the red solid line. The resonance plane at $\:z={L}_{r}$ is indicated by the red dashed line.

In contrast to a travelling plasma *wave*, a localized, plasma *oscillator* can be used as a standing-wave antenna that emits a narrowband THz radiation. Recently, a new idea of generating a radial plasma oscillator (RPO) was proposed^38^. The RPO can be formed in a density-gradient plasma region, where the beat-frequency of two slightly detuned laser pulses resonates with the local plasma frequency (see the schematic Fig. 1). While the conversion rate to THz wave from the plasma oscillator is considerably higher than that from the traveling plasma wave, as less of the laser energy is converted to the plasma oscillator, the net efficiency from the driving pulse to THz radiation remains similarly low as in plasma wave schemes. A straightforward way to overcome this limitation is to exploit the strong ponderomotive driving force of long-wavelength lasers, as has been emphasized in recent studies^39–41^. For a given field strength of the driving laser pulse, a more slowly oscillating field (i.e. longer wavelength) would transfer more momentum to the plasma electrons, leading to increased amplitude of plasma oscillation. Importantly, as the advances in mid-infrared optical parametric chirped-pulse amplification (OPCPA) and high-power CO₂ laser systems^42–44^ have already made such drivers experimentally available, such a new regime of plasma-based schemes for THz emission can be realized under realistic laboratory conditions. Recent studies have demonstrated that long-wavelength lasers, due to the much stronger ponderomotive force, can boost THz efficiency to 1–10% for broadband THz sources^45–47^. In this work, we use quasi-3D and 2D particle-in-cell simulations to show that employing mid-infrared drivers enhances the conversion efficiency to nearly 1%, which is unprecedented for plasma-based, *narrowband* THz sources. Importantly, we also validate the robustness of the mechanism under realistic plasma conditions, including field ionization and collisions, which confirm its feasibility for near-term experiments.

## Simulation results

### RPO with mid-infrared drivers

**Fig. 2:**
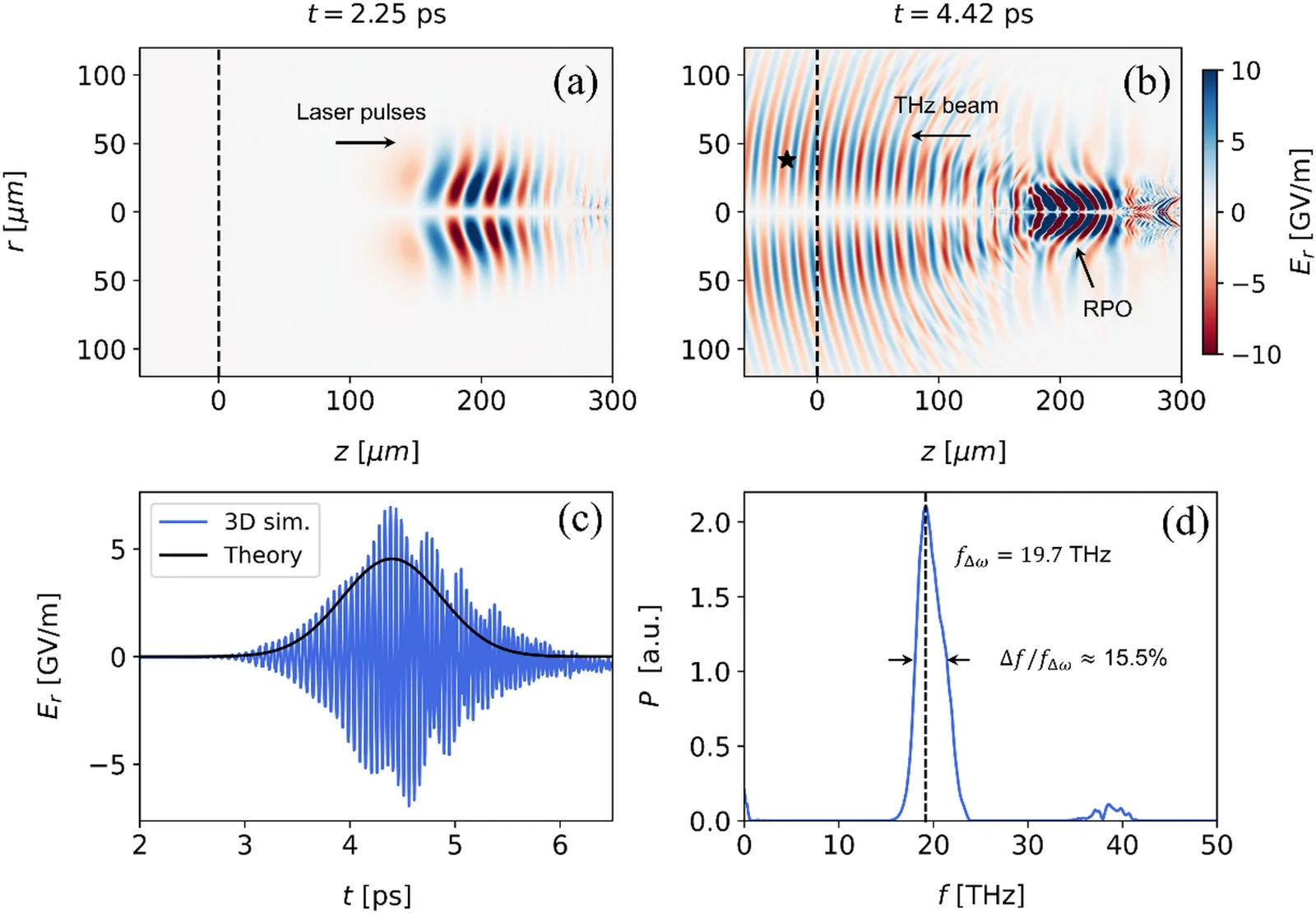
(**a**, **b**) Snapshots of radial electric field $\:{E}_{r}$, at $\:2.25\:$ ps, and * 4.42* ps. The black-dashed line represents the plasma vacuum boundary, and the black star indicates the position of a virtual point probe. (**c**) Time-evolution of the radial electric field obtained from the probe position (sky blue), and theoretical curve (solid black) given by Eq. (1). (**d**) Power spectrum corresponding to Fig. 2(**c**).

To verify a realistic RPO scheme based on mid-infrared lasers, we have performed quasi-3D particle-in-cell (PIC) simulations using the FBPIC code^48^. FBPIC provides accurate dispersion modelling that effectively suppresses artefacts such as numerical Cherenkov radiation. Moreover, it reproduces full 3D simulation results with high fidelity, while substantially reducing computational resource requirements. Details of the simulation methods are provided in the “Methods” section. It can be seen in Fig. 2a and b that the radiated THz wave is radially polarized and propagates in the backward direction with high directionality. A plasma wave packet is excited when the driving pulses pass the resonance point, as shown in Fig. 2a, and the THz radiation emitted from the plasma oscillator reaches a maximum when the plasma wave is localised in a single period (i.e., all electrons inside this wave packet oscillate in phase). To find the frequency and temporal profile of the THz pulse, we position a point probe at $\:z=-25\:{\,\upmu\mathrm{m}\:}$, $\:r=38\:{\,\upmu\mathrm{m}\:}$, and $\:\theta\:=0^{\circ}$. The temporal profile of the radiated field includes several cycles with a peak field strength of $\:{E}_{r}=6.94$ GV/m (Fig. 2c), which is in a good agreement with analytic calculations (solid black line) using Eq. (1). Its power spectrum has a single dominant peak, centred at the beat frequency of the laser pulses, $\:{f}_{\varDelta\:\omega\:}=\varDelta\:\omega\:/\left(2\pi\:\right)\approx\:$ 19.7 THz (where $\:\varDelta\:\omega\:={\omega\:}_{1}-{\omega\:}_{2}$ is the beat frequency of the driving laser pulses), with a relative spectral width of 15.5% at FWHM (full width at half maximum) (Fig. 2d), and a small peak at the second harmonic of the beat frequency $\:{f}_{2\left({\Delta\:}\omega\:\right)}\approx\:39.4$ THz. Radiation at high harmonics of the beat frequency can be generated at high laser intensity due to relativistic effects and wave breaking accompanied by plasma thermalization. However, the contribution of second harmonic radiation to the power spectrum is very small; thus, nearly all the THz beam energy is concentrated at the beat frequency (fundamental harmonic). Moreover, the spectral width can be reduced using longer duration driving pulses to localize the plasma oscillator more tightly^38^.

The energy of the emitted THz pulse and its conversion efficiency are estimated following Ref^38^. The power of the emitted THz radiation is obtained by the radial integration of the Poynting vector, $\:P\left(t\right)=2\pi\:{\int\:}_{0}^{\infty\:}S\:rdr$, where $\:S=\frac{1}{{\mu\:}_{0}} \left\langle {E_{r} B_{{\phi \:}} } \right\rangle$, the magnetic component of the THz radiation is $\:{\widehat{B}}_{\phi\:}\simeq\:\frac{1}{c}{\widehat{E}}_{r}$, and the radial component, $\:{\widehat{E}}_{r}$, is given by,

1 $$\:{\widehat{E}}_{r}\left(t\right)\simeq\:\frac{0.21{m}_{\mathrm{e}}c}{e}\frac{{a}_{0}^{2}{\Delta\:}{\omega\:}^{2}{\tau\:}_{\mathrm{L}}\sigma\:}{\sqrt{{r}_{\mathrm{L}}^{2}+4{\sigma\:}^{2}}}{h}^{2}\rho\:{\mathrm{e}}^{-h{\rho\:}^{2}}{\mathrm{e}}^{-4{t}^{2}/{\tau\:}_{E}^{2}}$$

where $\:{r}_{L}$, $\:{\tau\:}_{L}$ are the spot radius and duration of the driving laser pulses, respectively, and $\:{L}_{r}$ is the gradient scale length. $\:\rho\:=\sqrt{2}r/{r}_{L}$, $\:h={\left(1+i\xi\:/{z}_{R}\right)}^{-1}$, $\:\xi\:=\int\:{\eta\:}^{-1}dz$, the refractive index $\:\eta\:=\sqrt{z/{L}_{r}}\:$, and the Rayleigh length $\:{z}_{R}={\Delta\:}k{r}_{L}^{2}/4$, and $\:\sigma\:=4\sqrt{2}{L}_{r}/\left(\varDelta\:\omega\:{\tau\:}_{L}\right)$ is the longitudinal half-width of the plasma wave envelope. $\:{\tau\:}_{E}$ is the duration of the emitted THz radiation, which is close to $\:{\tau\:}_{E}=2{L}_{r}/c$. The THz energy is obtained by integrating $\:P\left(t\right)\:$ in time (see “Methods” section). With the driving laser pulse energy given by, $\:{U}_{L}={\left(\pi\:/2\right)}^{3/2}{({m}_{e}^{2}{c}^{2}{\omega\:}_{L}^{2}/{e}^{2}){\epsilon}_{0}a}_{0}^{2}c{\tau\:}_{L}{r}_{L}^{2}$, the energy conversion efficiency can be calculated as,

2 $$\:\eta\:={U}_{THz}/{U}_{L}\simeq\:0.0014\:{a}_{0}^{2}\varDelta\:{k}^{2}c\frac{{\tau\:}_{L}{L}_{r}}{1+{r}_{L}^{2}{\sigma\:}^{-2}/4}{\left(\frac{\varDelta\:\omega\:}{{\omega\:}_{L}}\right)}^{2}$$

where $\:\varDelta\:k={k}_{1}-{k}_{2}$ is the beat wavenumber of the driving pulses. Equation (2) indicates that the theoretical efficiency increases quadratically with the wavelength of the driving laser pulses, i.e. $\:\eta\:\propto\:{\lambda\:}_{L}^{2}$ for a fixed pulse energy ($\:\propto\:\:{a}_{0}^{2}{\tau\:}_{L})$. We verify this dependence through simulations for varying wavelengths, while keeping the parameters $\:{a}_{0},{\tau\:}_{L},$ and $\:\:{L}_{r}$ fixed.

**Fig. 3:**
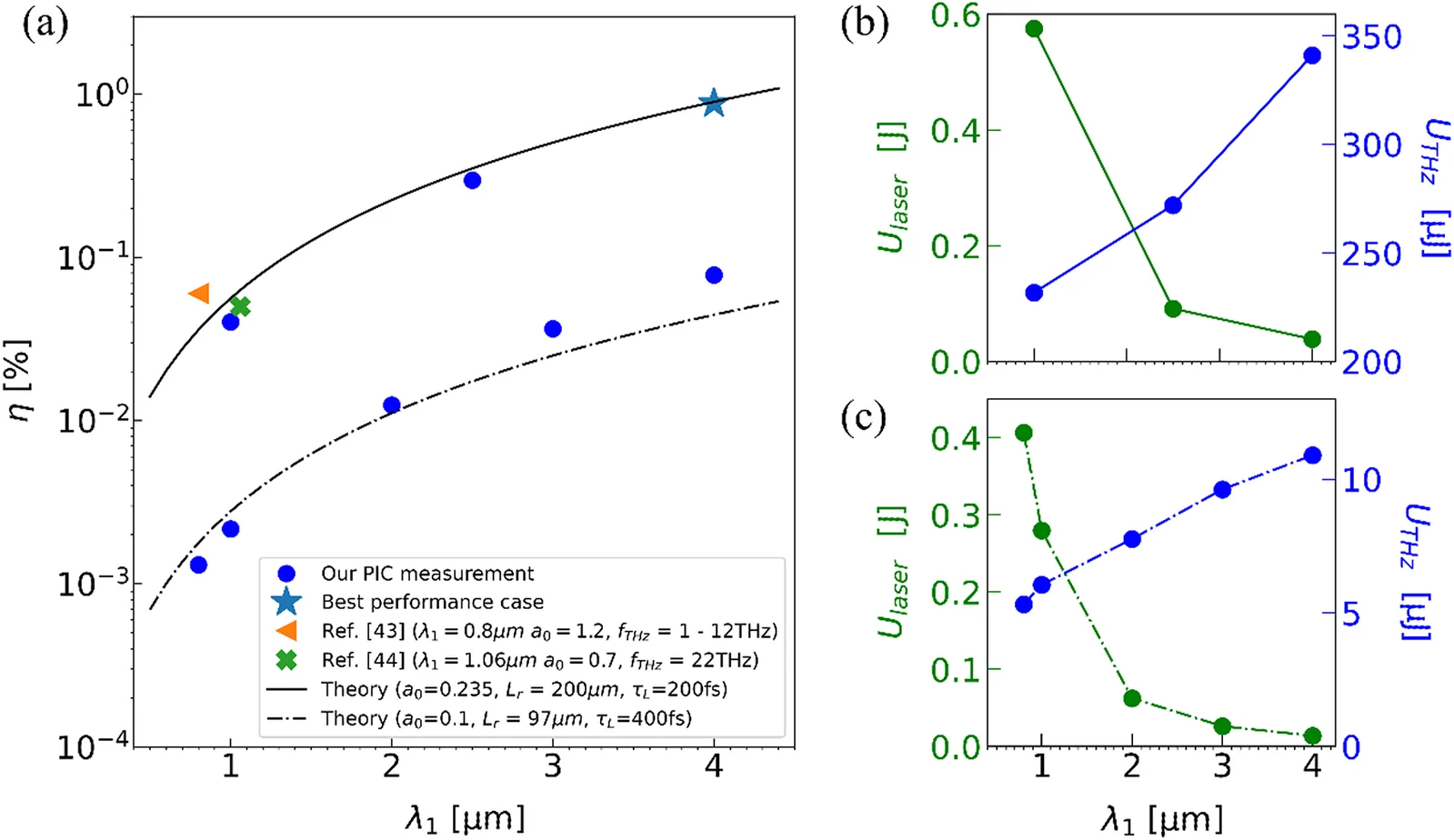
(**a**) The energy conversion efficiency as a function of the driving laser wavelength ($\:{\lambda\:}_{1}$) for the different simulation parameters. The *y*-axis is plotted on a logarithmic scale. The beat frequency of the driving lasers is set to be $\:{f}_{\varDelta\:\omega\:}\approx\:$ 19.7 THz. The black solid and dashed lines represent the theoretical curves from Eq. (2). Total laser energy and THz output energy as functions of the driving laser wavelength for (**b**) $\:{a}_{0}=0.235,\:\:{L}_{r}=200\;\upmu\mathrm{m},\:\:{\tau\:}_{L}=200\:\,\mathrm{f}\mathrm{s}$ and (**c**) $\:{a}_{0}=0.1,\:\:{L}_{r}=97\;\upmu\mathrm{m},\:\:{\tau\:}_{L}=400\:\,\mathrm{f}\mathrm{s}$.

Figure 3 shows that the simulated efficiency increases with increasing wavelength of the driving laser pulses, and reaches approximately 0.88%, in good agreement with the theoretical prediction using Eq. (2). For the best case, the total THz energy is approximately 0.341 mJ when the total laser energy is 0.0387 J. Compared with recently reported laser-to-THz efficiencies using optical wavelength lasers ($\:{\lambda\:}_{1}=0.8-1.06\:{\,\upmu\:}\mathrm{m}$) for the plasma-based narrowband THz sources^36,37^, which is typically of order $\:{10}^{-2}-{10}^{-1}\:$%, the RPO-based THz beam driven by a mid-infrared laser exhibits an efficiency enhanced by an order of magnitude.

### Realistic conditions: field ionization, ion motion, and Coulomb collisions

**Fig. 4:**
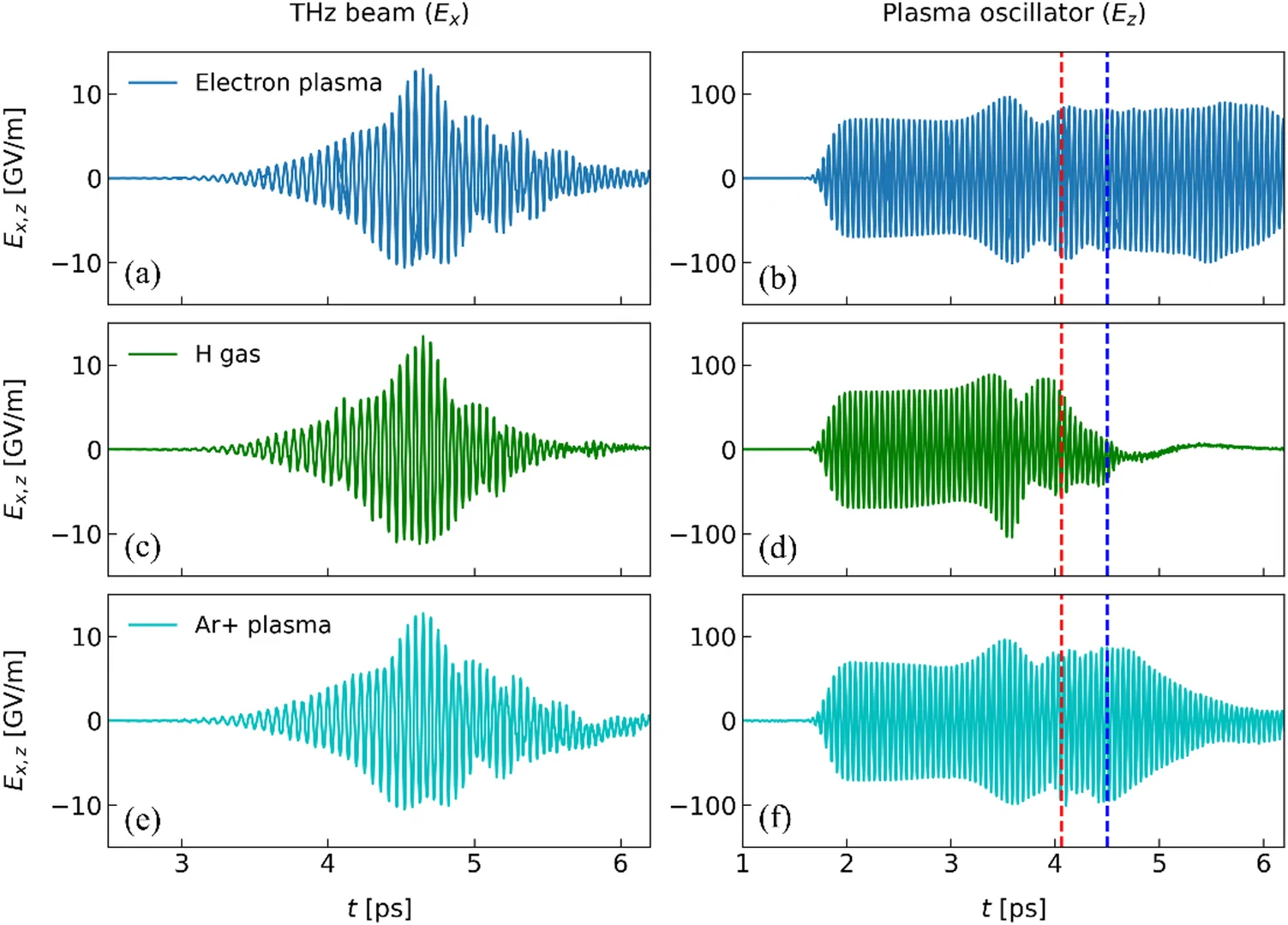
2D WarpX PIC simulation results; (**a**), (**c**), (**e**) temporal profiles of the THz electric field ($\:{E}_{x}$) and (**b**), (**d**), (**f**) longitudinal electric field ($\:{E}_{z})$ of plasma oscillator from (**c**), (**d**) neutral hydrogen gas and (**e**), (**f**) singly-ionized argon plasma, compared to (**a**), (**b**) the preloaded electron plasma. Virtual probes are located at $\:z=-25\:{\,\upmu\:}\mathrm{m},\:\:x=38\:{\,\upmu\:}\mathrm{m}$ for recording the THz electric field in vacuum, and $\:z=200\:{\,\upmu\:}\mathrm{m},\:\:x=0\:{\,\upmu\:}\mathrm{m}$ for recording the longitudinal electric field near the resonance position. The red dashed line indicates the beginning of the THz burst, while the blue dashed line indicates its termination.

The model of the RPO is extended using simulations under more realistic conditions, which include field ionization of neutral gas and collisional effects. The simulations were conducted using the same parameters as in Fig. 2 with these effects incorporated. The PIC code WarpX^49^ has been used with the Pérez’s Monte-Carlo binary collision model^50^. Only electron-ion collisions have been considered because same-species collisions do not change the net fluid momentum and are ineffective in energy dissipation. Field ionization in WarpX is based on the Ammosov–Delone–Krainov (ADK) model^51,52^. For the ADK model, the ionization dynamics generally depend on the wavelength of the driving laser. The Keldysh parameter is defined as $\:{\gamma\:}_{K}={\omega\:}_{0}\sqrt{2{m}_{e}{I}_{p}\:}/e{E}_{L}=\sqrt{2{I}_{p}/{m}_{e}{c}^{2}}\:{a}_{0}^{-1}\:\:$where $\:{\omega\:}_{0}=2\pi\:c/{\lambda\:}_{0}$ is the laser angular frequency, $\:{\lambda\:}_{0}$ is the laser wavelength in vacuum, $\:{E}_{L}$ is the peak field strength of the laser pulse, and $\:{I}_{p}$ is the ionization energy. For the parameters used in our simulations for hydrogen, when $\:{a}_{0}$ is fixed, we obtain $\:{\gamma\:}_{K}\ll\:1$, indicating that ionization occurs in the tunneling regime. Consequently, within the parameter range considered in this study, wavelength-dependent ionization dynamics are not expected to significantly affect the plasma formation up to the resonance point. Only two-dimensional (2D) simulations have been carried out due to limited computational resources. Figure 4a–f shows the temporal profiles of the electric fields of the THz emission and the RPO for initially neutral hydrogen and a pre-formed single-ionized argon plasma. For comparison, a pre-formed electron plasma case with fixed ion background is also presented. For hydrogen gas, the THz beam energy is reduced by about 40% compared with the pre-formed electron plasma case, primarily due to the early decay of the emission field. Figure 4c shows that the transverse field strength of the THz beam in hydrogen gas decreases rapidly after 5.25 ps, whereas in pre-formed electron plasma it persists for a much longer time (Fig. 4a). This early decay in the hydrogen case is explained by excitation of ion motion, which takes longitudinal field energy from the RPO. Consistently, Fig. 4b shows that for pre-formed electron plasma, the plasma oscillator field remains quasi-constant between 4.06 ps and 4.5 ps (indicated by the vertical red and blue lines), while, in hydrogen gas, strong ion motion weakens the longitudinal field strength of the RPO, leading to a decay of the THz burst. We explored the use of a heavier ion species to overcome this issue; for a pre-formed, single-ionized argon plasma, with ion-electron collision effects being incorporated, the initial electron (ion) temperature is set to $\:{T}_{e}=300\:$ eV($\:{T}_{i}=0.3\:$ eV). With those conditions, the transverse field strength of the THz beam is comparable to that in the pre-formed electron plasma (Fig. 4e). The THz beam energy dissipation into ion motion in this case is only 8%, which is considerably smaller than that observed in hydrogen gas. Although the longitudinal field strength of the plasma oscillator begins to decrease after the time *t* = 4.5 ps, it does not affect the efficiency as the emission for * t>4.5* ps is negligibly small for both electron plasma and Ar plasma cases. The THz energy conversion efficiency inferred from the 2D simulations reaches 0.87% for single-ionized argon plasma and 0.57% for neutral hydrogen gas, compared with 0.95% for the pre-formed electron plasma.

**Fig. 5:**
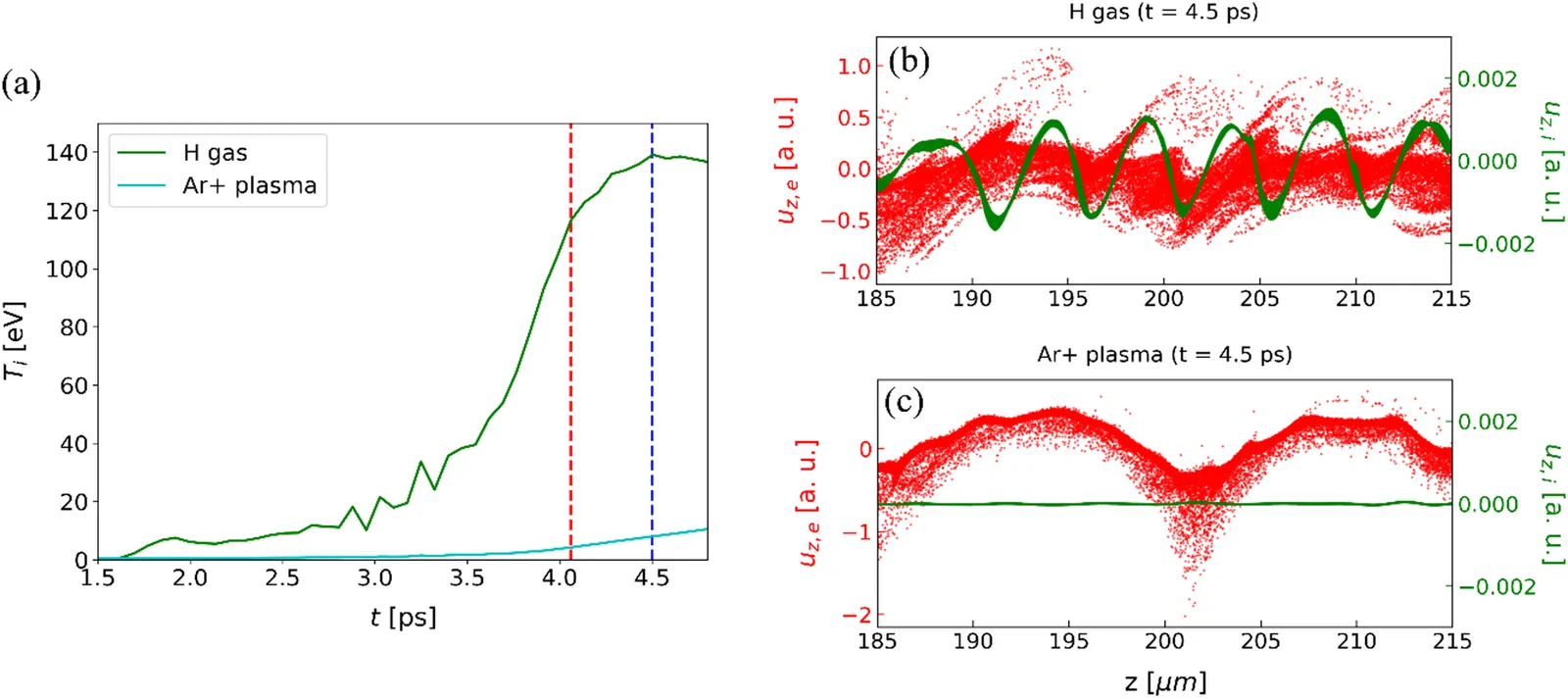
(**a**) Time-evolution of ion-temperature for neutral hydrogen gas and singly-ionized argon plasma. The ion-temperature is calculated as average in the region $\:z\in\:\left[170\:{\,\upmu\:}\mathrm{m},\:\:230\:{\,\upmu\:}\mathrm{m}\right],\:x\in\:[-30\:{\,\upmu\:}\mathrm{m},\:30\:\,{\upmu\:}\mathrm{m}]$. The red and blue dashed lines indicate the same times as in Fig. 4. Longitudinal phase space ($\:{u}_{z}$) of electrons and ions near the resonance point for (**b**) an ionized H⁺ plasma from hydrogen gas and (**c**) an Ar⁺ plasma at $\:t=4.5\:$ps.

To confirm that the ion motion within the plasma wave packet dampens the electron oscillations in the RPO and therefore suppresses the THz emission, we have calculated the time-evolution of the space-averaged ion-temperature within the RPO. As shown in Fig. 5a, the ion temperature for the hydrogen case reaches $\:{T}_{i}\:\sim\:130$ eV after 4.28 ps, the time when the electron oscillation has decayed substantially. This is approximately 10 times higher than in singly-ionized argon plasma, $\:{T}_{i}\:\sim\:13$ eV, strongly indicating that the rapid damping of the longitudinal electric field of the RPO in Fig. 5d results from excitation of ion motion. Furthermore, at $\:t=4.5\:$ ps we observe excitation of a considerably larger longitudinal ion wave near the resonance point in field-ionized hydrogen plasma, compared with singly-ionized argon plasma, as shown in Fig. 5b and c. All those effects lead to the disruption of the oscillation of the RPO, playing as a critical factor in the energy reduction of the THz beam.

When binary Coulomb collisions are included in the initially neutral hydrogen gas and single-ionized argon plasma cases, no significant effect on the RPO is observed, because the electron-ion collision frequency is much lower than the plasma oscillation frequency. For example, in singly-ionized argon plasma, the electron-ion collision frequency is $\:{\nu\:}_{ei}\approx\:2.8\times\:{10}^{10}$ Hz using the relation $\:\:{\nu\:}_{ei}\:\left[\mathrm{H}\mathrm{z}\right]\simeq\:3\times\:{10}^{-5}{n}_{e}\left[\mathrm{c}{\mathrm{m}}^{-3}\right]{\left({T}_{e}\left[\mathrm{e}\mathrm{V}\right]\right)}^{-3/2}$, where $\:{T}_{e}=300$ eV, and $\:{n}_{e}=4.85\times\:{10}^{18}\:\mathrm{c}{\mathrm{m}}^{-3}$ (at the resonance point), and the Coulomb logarithm is $\:\mathrm{ln}{\Lambda\:}=10$^53^. A simple estimate yields $\:{\nu\:}_{ei}/{f}_{{\Delta\:}\omega\:}\approx\:$ 0.142%, implying that the influence of the collisions on the rapid electron oscillations of RPO is fairly small. Multiplying the THz burst period with the electron-ion collision frequency yields a collisional energy dissipation of approximately 2.1%, which is only a small effect. While realistic neutral gas dynamics involve complex collisional effects, such as electron-neutral collisions, collisional ionization, and excitation, these processes were excluded from our simulations due to computational resources. Nevertheless, because the laser intensity is high enough to drive full ionization, the ratio $\:{\nu\:}_{en}/{f}_{{\Delta\:}\omega\:}$ (where $\:{\nu\:}_{en}$ is electron-neutral collision frequency) remains exceedingly small, ensuring a negligible impact on the RPO-THz radiation.

## Conclusion

In conclusion, in the system of THz radiation from RPO (radial plasma oscillator) driven by mid-infrared laser pulses, we achieved an energy conversion efficiency of nearly 1%, which is three-orders of magnitude higher than our previous result obtained at optical wavelengths ($\sim 0.8\, \:{\upmu\:}\mathrm{m}$). Under optimised conditions, a 0.341 mJ, 19.7 THz radiation with a spectral width of 15.5%, and a peak field strength of 6.94 GV/m can be produced. For field-ionized hydrogen plasma, studied using 2D WarpX, THz beam energy losses reach up to 40%, compared with pre-formed electron plasma with fixed ion background. As the lost energy is taken by the ion motion, the energy loss can be mitigated by using heavier ion species. For a pre-formed single-ionized argon plasma, the THz beam energy loss is 8%, which is very small compared to the hydrogen gas case. We verified by simulations and theoretical estimation that the effects of electron-ion Coulomb collisions are negligible. The RPO-based THz source driven by longer wavelength lasers can generate narrowband, sub-millijoule-level THz radiation under realistic conditions. The high-efficiency narrowband THz can be a competitive option for various high-power THz applications, such as THz-band compact accelerators.

## Methods

### Numerical simulations

In the quasi-3D FBPIC simulations, we considered two detuned, copropagating Gaussian pulses, with frequencies $\:{\omega\:}_{1}=2\pi\:c/{\lambda\:}_{1}$ and $\:{\omega\:}_{2}=2\pi\:c/{\lambda\:}_{2}$, where $\:{\lambda\:}_{1}=4.0\:{\,\upmu\:}\mathrm{m}$, $\:{\lambda\:}_{2}=5.416\:{\,\upmu\:}\mathrm{m}$. The normalized vector potential of both laser pulses, $\:{a}_{0}={a}_{\mathrm{1,2}}=0.235=e{E}_{L}/\left({m}_{e}c{\omega\:}_{\mathrm{1,2}}\right)$, where $\:{E}_{L},\:\:{m}_{e},\:\:c$ and * e* are the laser electric field, electron mass, speed of light and elementary charge, respectively. The spot radius is $\:{r}_{L}=40\:{\,\upmu\:}\mathrm{m}$ and the pulse duration $\:{\tau\:}_{L}=200\:$ fs, where $\:{E}_{L}\propto\:\mathrm{e}^{-{r}^{2}/{r}_{L}^{2}}\mathrm{e}^{-{\left(z-ct\right)}^{2}/({c}^{2}{\tau\:}_{L}^{2})}$. Gaussian laser pulses, truncated at $\:3{\tau\:}_{L}$, are launched at * t=0* from the left boundary. The plasma slab has a linear density gradient up-ramping from 0 to 0.$\:1{n}_{c}$, where $\:{n}_{c}={\epsilon}_{0}{m}_{e}{\omega\:}_{1}^{2}/{e}^{2}$ is the critical density for $\:{\omega\:}_{1}$ and ${\epsilon}_{0}$ is the vacuum permittivity, over a distance of $0-\:285\:{\,\upmu\:}\mathrm{m}$, followed by a uniform density region extending to $\:300\:{\upmu\:}\mathrm{m}$. The distance at which the beat frequency of laser pulses matches the plasma frequency $\:{\omega\:}_{pe}\left(z\right)$, defined by a gradient scale length $\:{L}_{r}$, equals to $\:200\:{\,\upmu\:}\mathrm{m}$. We used two azimuthal modes to implement the linear polarization of the driving laser pulses, and the simulation domain is $\:360\times\:120\:{\,\upmu\:}{\mathrm{m}}^{2}$, in * z* and * r* directions, which are discretized into 7200 and 1200 cells, respectively. The same parameters have been used in 2D WarpX and FBPIC simulations. For the 2D WarpX simulations, the * x* range was defined as twice the * r* range of the FBPIC simulations.

### Calculation of RPO-THz energy

In Fig. 2, to calculate the THz beam energy, we performed the integration in cylindrical coordinates, as given by the following equation:

$${U}_{THz}=\int\limits^{\infty\:}_{-\infty}P\left(t\right)dt=\int\limits_{-\infty\:}^{\infty\:}\int\limits_{0}^{2\pi\:}\int\limits_{0}^{\infty}\frac{1}{2}c{\epsilon}_{0}{E}_{r}^{2}rdrd\theta\:dt\:$$

where $\:P\left(t\right)$ is total power, which was evaluated at $\:z=-30\,\mu\:m$ over the entire temporal range. The integration range for *r* is $\:\left[0\:{\,\upmu\:}\mathrm{m},\:\:120\:{\,\upmu\:}\mathrm{m}\right]$, assuming azimuthal symmetry and total laser energy was also computed in the same way. For 2D WarpX simulation, we estimated the THz energy using a line probe at z at $\:z=-10\,\:\upmu\:\mathrm{m}$ over the entire temporal range. The corresponding integration range for *x* is $\:\left[-120\:{\,\upmu\:}\mathrm{m},\:\:120\:{\, \upmu\:}\mathrm{m}\right]$ and the integral in the cartesian coordinates system was calculated using the following equation.

$$\frac{d{U}_{THz}}{dy}=\int\limits_{-\infty\:}^{\infty\:}\int\limits_{0}^{\infty}\frac{1}{2}c{\epsilon}_{0}{E}_{x}^{2}dt\:dx\:$$

In both the quasi-3D and 2D simulations, measurements were taken in the far-field region, with the distance from the RPO to the observation point is satisfied $\:r\gg\:{\lambda\:}_{pe}$ where $\:{\lambda\:}_{pe}$ is the local plasma wavelength of THz radiation.

## Data Availability

The data supporting this study’s findings are available from the corresponding author upon reasonable request.
